# Genetic Divergence of Vibrio vulnificus Clinical Isolates with Mild to Severe Outcomes

**DOI:** 10.1128/mbio.01500-22

**Published:** 2022-09-28

**Authors:** Kendall Kling, Sonya A. Trinh, Semen A. Leyn, Dmitry A. Rodionov, Ivan D. Rodionov, Alfa Herrera, Kasey Cervantes, George Pankey, Deborah Ashcraft, Egon A. Ozer, Adam Godzik, Karla J. F. Satchell

**Affiliations:** a Division of Infectious Diseases, Department of Medicine, Northwestern University Feinberg School of Medicine, Chicago, Illinois, USA; b Division of Infectious Diseases, Ochsner Medical Center, New Orleans, Louisiana, USA; c Sanford Burnham Prebys Medical Discovery Institute, LaJolla, California, USA; d University of California, San Diego, La Jolla, California, USA; e Infectious Disease Translational Research, Ochsner Clinic Foundation, New Orleans, Louisiana, USA; f Biosciences Division, University of California Riverside School of Medicine, Riverside, California, USA; g Department of Microbiology-Immunology, Northwestern University Feinberg School of Medicine, Chicago, Illinois, USA; Fred Hutchinson Cancer Center

**Keywords:** *Vibrio vulnificus*, genome, phylogeny, toxin, surveillance, RTX toxins, patient outcome

## Abstract

The marine bacterium Vibrio vulnificus infects humans via food or water contamination, leading to serious manifestations, including gastroenteritis, wound infections, and septic shock. Previous studies suggest phylogenetic Lineage 1 isolates with the v*cgC* allele of the *vcg* gene cause human infections, whereas Lineage 2 isolates with the *vcgE* allele are less pathogenic. Mouse studies suggest that some variants of the primary toxin could drive more serious infections. A collection of 109 V. vulnificus United States human clinical isolates from 2001 to 2019 with paired clinical outcome data were assembled. The isolates underwent whole-genome sequencing, multilocus-sequence phylogenetic analysis, and toxinotype analysis of the multifunctional autoprocessing repeats-in-toxin (MARTX) toxin. In contrast to prior reports, clinical isolates were equally distributed between lineages. We found no correlation between phylogenetic lineage or MARTX toxinotype and disease severity. Infections caused by isolates in Lineage 1 demonstrated a borderline statistically significant higher mortality. Lineage 1 isolates had a trend toward a higher proportion of M-type MARTX toxins compared with Lineage 2, although this was not statistically significant.

## INTRODUCTION

Vibrio vulnificus is a Gram-negative halophilic bacterium that is part of the natural flora of warm coastal environments, but is also an opportunistic pathogen that causes life-threatening foodborne and wound infections worldwide (1). Foodborne infections occur after the ingestion of raw or undercooked seafood, particularly oysters, and result in illnesses ranging from a self-limited gastroenteritis to septicemia with rapid progression to multi-organ failure and death (2). Wound infections occur after seawater or seafood penetrates the skin layer and can spread to deeper structures, leading to necrotizing fasciitis requiring surgical debridement and even amputation (3). Several underlying medical conditions have been identified as risk factors for V. vulnificus infection, including chronic liver disease, diabetes mellitus, kidney disease, autoimmune disease, hematological disorders, and malignancy (4).

The incidence of V. vulnificus infections remains low despite the high proportion of patients at risk for infection and the widespread distribution of the pathogen in warm marine environments. Despite the fact that an estimated 19 to 27 million individuals are at risk for V. vulnificus infection, the incidence of reported infections is low, with the average number of cases in the United States at 120 per year (5). Raw oyster consumers account for 8% to 34% of the population, depending on the region, and oysters contain 10^3^ to 10^4^ CFU of V. vulnificus per gram during summer months (5). The discrepancy between the at-risk population and disease incidence suggests that either host factors or intrinsic bacterial factors contribute to disease severity. Identification of pathogenic strains in seafood harvests and coastal environments could reduce the incidence of V. vulnificus foodborne and wound infections.

Many studies have attempted to identify genetic markers that would predict which V. vulnificus strains can cause human disease with severe outcomes (6). Initially, strains were classified into three biotypes based on biochemical characteristics: Biotype 1 implicated in the majority of human infections, Biotype 2 primarily an eel pathogen, and Biotype 3 responsible for wound infections linked to tilapia contact in Israel (7). Additional studies have suggested specific determinants may distinguish pathogenic strains, including 16S rRNA, the *pilF* gene, and mannitol fermentation, but these characteristics also have been found among environmental strains (8, 9).

Multilocus sequence analysis (MLSA) of Biotype 1 strains revealed these strains split into two genetic lineages as predicted by the nucleotide sequence of the virulence-correlated gene (*vcg*), with clinical strains clustering in Lineage 1 (*vcgC*) and environmental strains represented in Lineage 2 (*vcgE*) (10). Sequence analysis of core genomes further identified a total of five lineages that did not correspond with the previously described biotypes, and found that most human clinical isolates clustered in Lineage 1 (11). As a result of these findings, rapid amplification methods to distinguish *vcgC* and *vcgE* alleles have been utilized in environmental and food safety research to monitor pathogenic V. vulnificus populations in coastal waters and harvests (12, 13).

However, there have been studies that challenge this dogma that isolates with potential to cause human disease would cluster in Lineage 1. A mouse model study of environmental and clinical strains found that isolates with the *vcgC* allele were more common in clinical strains and were more likely to cause severe infection and death, but isolates with the *vcgE* allele also were capable of causing lethal infections (14). Another mouse study utilized strains both from clinical and environmental sources and found no difference in virulence between *vcgC* and *vcgE* strains, although isolates obtained from colder temperature seasons were more virulent (15). Thus, despite many attempts to categorize V. vulnificus, the predictive value of genetic analysis, biochemical assays, and phylogeny to predict pathogenic potential remains unclear.

**FIG 1:**
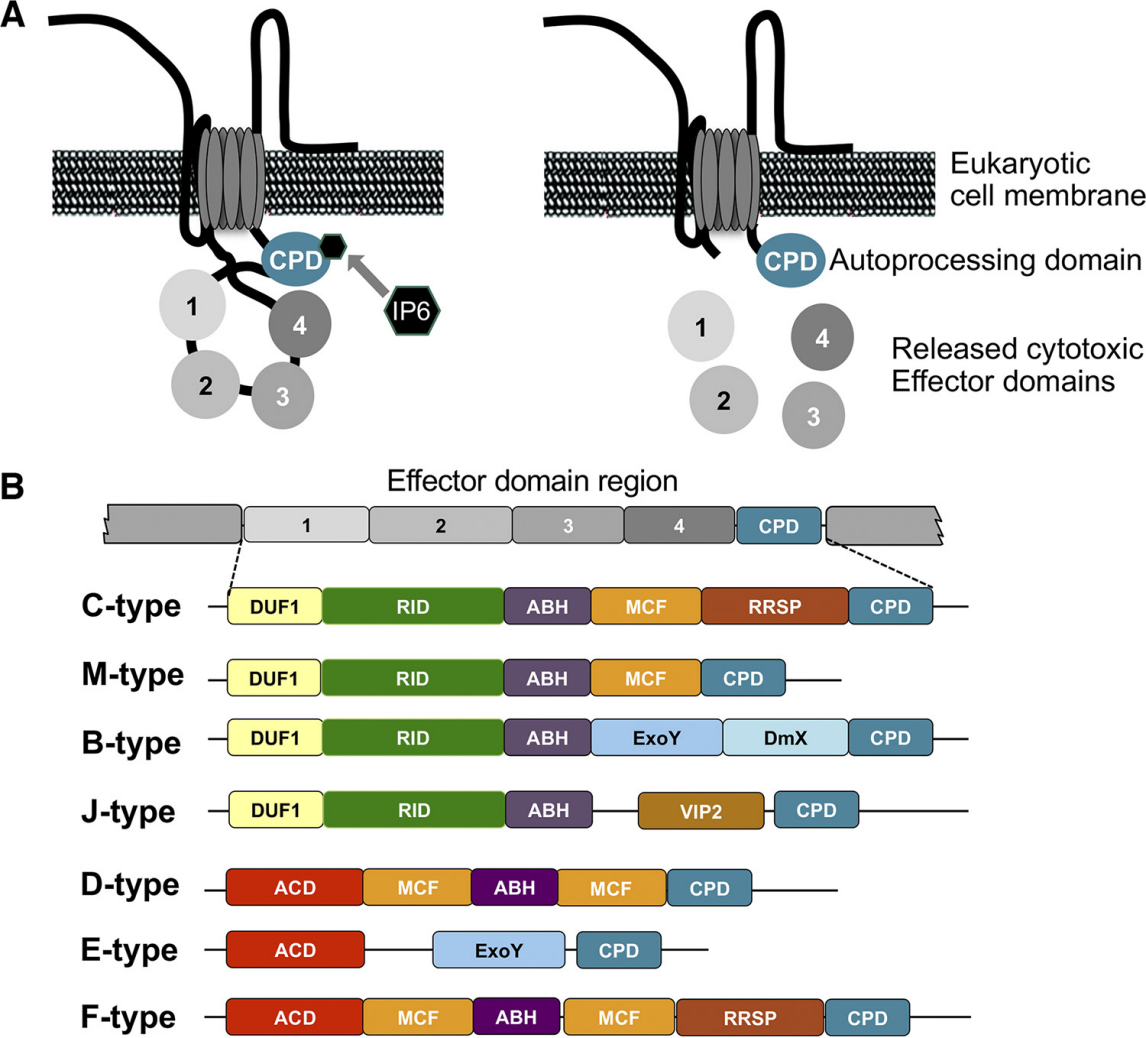
MARTX toxin structure, the main virulence factor of Vibrio vulnificus. (A) Diagrammatic depiction of mechanism of toxin entry into eukaryotic cells. Following membrane insertion, the central portion of the toxin is translocated across the membrane. Autoprocessing by the cysteine protease domain (CPD) is initiated by binding of inositol hexakisphosphate (IP6), resulting in release of effector domains. (B) The effector domains are variable in different strains. The toxins are assemblages of effectors: DUF1, domain of unknown function in the first position; RID, rho inactivation domain; ABH, alpha-beta hydrolase domain; MCF, makes caterpillars floppy-like cysteine protease; RRSP, Ras-Rap1-specific endopeptidase; ExoY, Pseudomonas ExoY-like adenylate cyclase; DmX, domain X cysteine protease; VIP2, vegetative insecticidal protein 2-like domain; ACD, actin cross-linking domain. For detailed descriptions of MARTX toxins and the effector domains, see Gavin and Satchell, 2015 (43); Woida and Satchell, 2018 (22); and Kim, 2018 (23).

One difference that has also been suggested to predict virulence potential is the composition of the primary virulence toxin. The multifunctional autoprocessing repeats-in-toxin (MARTX) is the primary virulence factor for V. vulnificus infection in mouse models (16 to 19). The MARTX toxin forms pores in the eukaryotic cell plasma membrane and releases effector domain proteins into the cytoplasm, where they can perform a variety of cytotoxic activities, including necrosis, apoptosis, actin depolarization, activation of reactive oxygen species, and suppress the host immune response. (Fig. 1A) (20 to 23). Among V. vulnificus isolates, there are multiple MARTX toxin variants each with a unique repertoire of effector domains (24, 25) (Fig. 1B). Mouse models of V. vulnificus infection have suggested that overall virulence can be dependent upon the MARTX toxinotype, with C-type toxins more potent than M-type or B-type variants (24, 26, 27). However, it is unknown whether MARTX variants can explain the range of clinical presentations in human V. vulnificus infections.

Overall, it is unknown if severity of clinical disease correlates with phylogenetic lineage or MARTX type, as no prior studies have been performed that pair genome analysis with patient outcome data. The objective of the study was to use whole-genome sequencing analysis of clinical V. vulnificus isolates with documented clinical outcomes to assess the relationships among V. vulnificus genetic background, MARTX variants, and infection severity.

## RESULTS

### Cohort of U.S. V. vulnificus clinical isolates from 2001 to 2019 with paired patient and clinical outcomes data.

From 2007 through 2013, there were 114 V. vulnificus isolates submitted to the CDC that could be matched with Cholera and Other Vibrio Illness Surveillance (COVIS) reporting forms. Microbiological and genome-based identification revealed that several isolates were other species, including Vibrio parahaemolyticus, Vibrio cholerae, Vibrio mimicus, and Aeromonas veronii. Of the remaining 104 V. vulnificus isolates from the original batch, 73 passed quality control measures after whole-genome sequencing. From the second patient pool from 2001 to 2019, 42 isolates were obtained to increase the representation of Gulf States in the collection. Forty isolates were included in the collection as 2 were subsequently identified as other bacterial species and only 36 had corresponding clinical data. Clinical data without paired isolates were obtained for 2,413 suspected V. vulnificus cases from the same time period of 2001 to 2019.

Therefore, the isolate collection for this study comprised a total of 109 V. vulnificus isolates with corresponding clinical data (Table S1). Patient characteristics were age, sex, liver disease, alcohol abuse, and presence of other comorbidities (cardiovascular disease, diabetes, renal disease, or malignancy). Infection type was characterized as either wound or gastrointestinal based on available clinical data.

10.1128/mbio.01500-22.1 TABLE S1 Supplemental data set. Demographic information on clinical isolates of Vibrio vulnificus in this study. Download Table S1, XLSX file, 0.02 MB. Copyright © 2022 Kling et al. 2022 Kling et al. https://creativecommons.org/licenses/by/4.0/ This content is distributed under the terms of the Creative Commons Attribution 4.0 International license.

The 109 sequenced isolates had no significant difference in patient characteristics compared to all reported cases for 2001 to 2019 (Table 1). Among the patients with a sequenced V. vulnificus isolate, the mean age was 59 years (range 5 to 87), and 93% were male. Alcoholism was reported in 27% of patients, and liver disease in 34% of patients. Gastrointestinal infections occurred in 39% of patients, and wound infection in 60%. Patients with adverse clinical outcomes include 11% requiring hospitalization for longer than 14 days, 14% with amputation, and 30% with death. The time to death ranged from 1 to 59 days. It appeared that rate of gastrointestinal versus wound infection type had a statistically significant difference; however, for 59% of all isolates reported to COVIS, a definitive source was not available or known. The isolates we sequenced had significantly more amputations and hospital stay greater than 14 days compared to all patients reported to COVIS, but there was no significant difference in mortality.

**TABLE 1:** Patient characteristics, clinical presentation, and outcomes associated with sequenced V. vulnificus isolates compared to all V. vulnificus infections reported to COVIS from 2001 through 2019

Demographic information	Isolates with paired clinical data (*n *= 109)	Total reported to COVIS (*n* = 2,431)	*P* value
Patient characteristics			
Age, yrs [avg (range)]	59 (5–87)	60 (0–100)	0.68
Male (%)	101 (93)	2,038 (84)	0.32
Liver disease	37 (34)	680 (28)	0.23
Alcohol	29 (27)	583 (24)	0.56
Other comorbidities	70 (64)	1,681 (69)	0.56
State (*n*)	Louisiana (33) Texas (27) Georgia (12) Maryland (11) Tennessee (5) North Carolina (4) Virginia (4) Hawaii (3) Arizona (2) Florida (2) Alabama (1) Connecticut (1) Illinois (1) Massachusetts (1) Ohio (1) Rhode Island (1)	Florida (577) Texas (430) Louisiana (233) Maryland (170) Virginia (144) Alabama (101) Georgia (96) North Carolina (91) Mississippi (85) Hawaii (68) California (62) Tennessee (45) New Jersey (43) New York (42) South Carolina (38) Arizona (24) Pennsylvania (17) Colorado (14) Connecticut (12) Massachusetts (12) Michigan (12) Oklahoma (12) Delaware (10) Indiana (8) Kentucky (8) Ohio (8) Illinois (7) District of Columbia (6) Minnesota (6) New Mexico (6) Rhode Island (6) Maine (5) Arkansas (4) Kansas (4) Missouri (4) Washington (4) Nevada (3) Oregon (3) Wisconsin (3) New Hampshire (2) Utah (2) Guam (1) Nebraska (1) North Dakota (1) Vermont (1)	
Infection type			
Gastrointestinal (%)	42 (39)	547 (23)	0.0006
Wound (%)	65 (60)	870 (36)	0.00003
Other or unknown (%)	2 (1)	1,014 (42)	
Outcomes			
Hospitalization >14 days (%)	12 (11)	145 (6)	0.03
Amputation	15 (14)	126 (5)	0.00004
Death	33 (30)	590 (24)	0.18
Time to death, days (range)	1–59	0–123	

**FIG 2:**
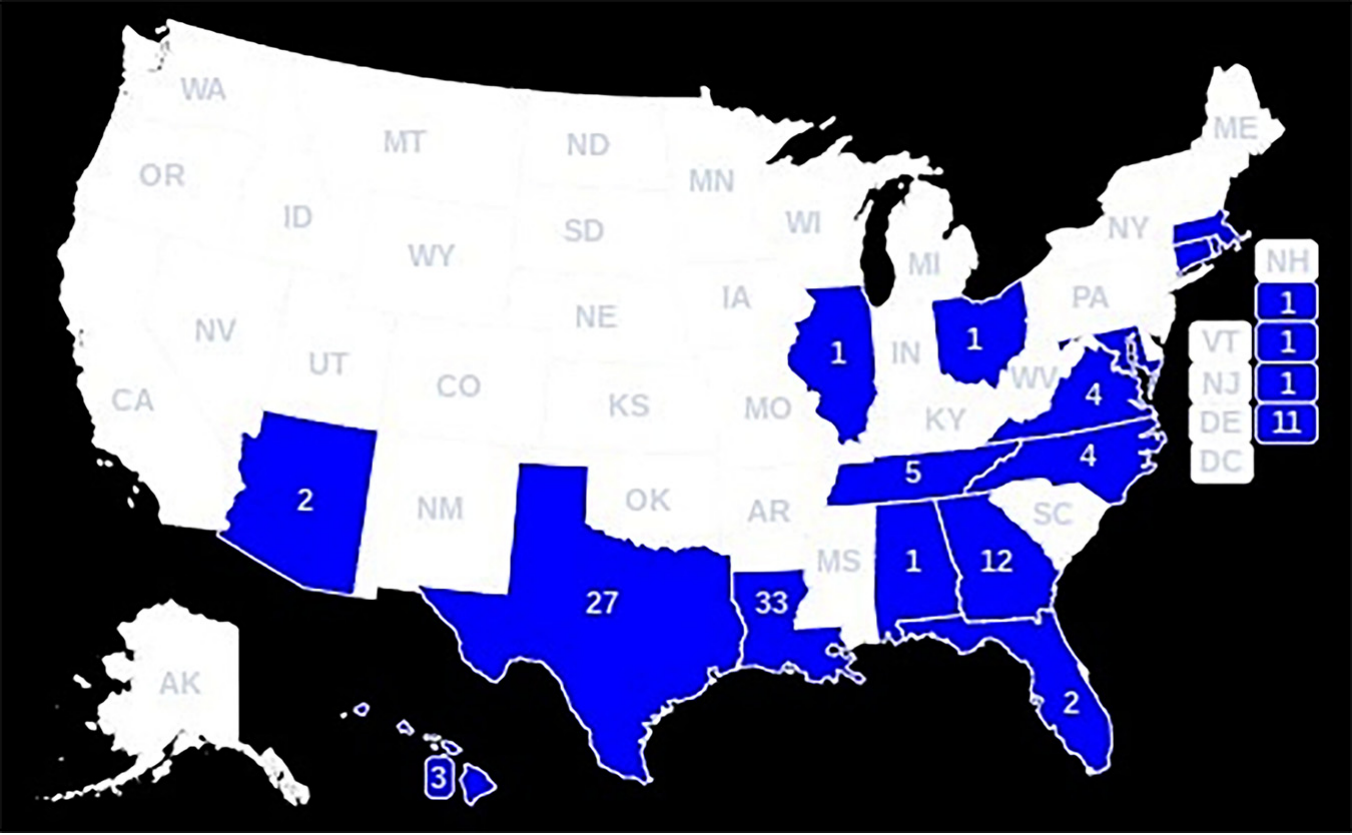
Map of geographic origin of 109 sequenced Vibrio vulnificus isolates analyzed in this study. Diagram was generated at simplemaps.com.

For all COVIS-reported cases from 2001 to 2019, the states with the highest prevalence of reported V. vulnificus infections were Florida, Texas, and Louisiana. Among reported cases with linked isolates from 2001 to 2019, the states with the most V. vulnificus infections included Louisiana, Texas, Georgia, and Maryland (Fig. 2).

**FIG 3:**
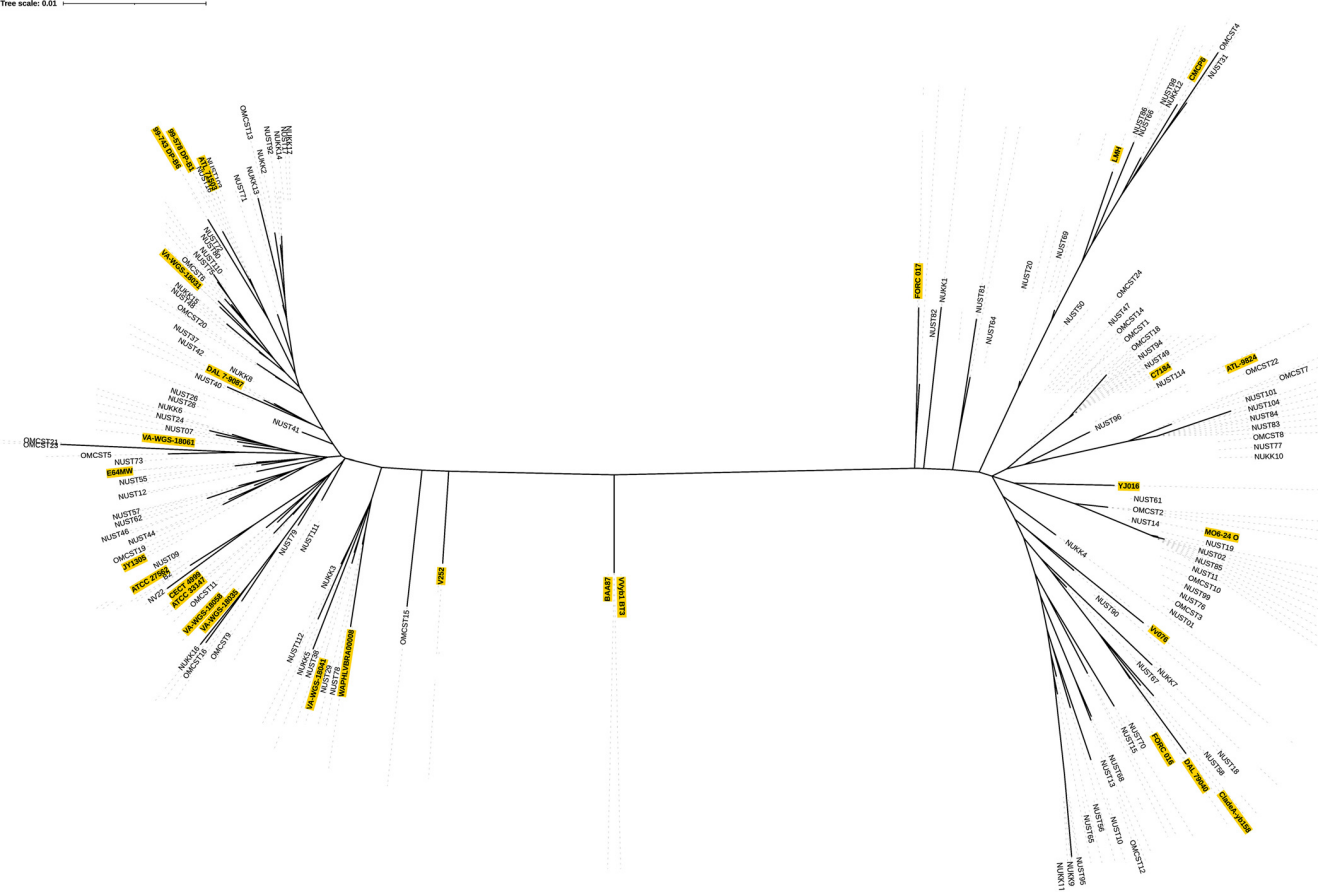
Unrooted MLSA phylogenetic tree of clinical isolates of V. vulnificus with reference isolates. Maximum likelihood phylogeny of concatenated DNA sequences for 10 housekeeping genes extracted from whole-genome sequences according to the standard MLSA method of Bisharat et al. (29). MLSA sequences for representative isolates obtained from the PubMLST database (highlighted in yellow) were used to identify Lineages 1 and 2 and sublineages. Diagram was generated in iTOL (42).

We assembled a maximum likelihood phylogenetic tree of clinical isolates and reference genomes using the standardized 10-locus MLSA method of Bisharat et al. (28). The unrooted phylogenetic tree (Fig. 3) revealed the clinical isolates split into two distinct lineages. Reference genomes from prior studies were included to identify branches. These are referred to here as Lineage 1, also known as Clade 1 or Lineage C and previously closely linked with clinical isolates, and Lineage 2, also known as Clade 2 or Lineage E and closely linked with environmental isolates. PCR identification of the *vcg* gene allele in the first pool and analysis of DNA sequence in the second pool confirmed Lineage 1 isolates have the *vcgC* allele sequence while Lineage 2 carries the *vcgE* allele. No sequenced genomes from this collection clustered with Biotype 3 reference genomes VVby1 or BAA87, indicating our collection did not include any Biotype 3 isolates, consistent with these isolates being geographically restricted to Israel (7, 28).

### Severe disease outcome does not correlate with lineage.

Patient characteristics of Lineage 1 and Lineage 2 strains were compared (Table 2). There was no significant difference in gastrointestinal versus wound infections between the lineages. While there was no significant difference in hospitalization >14 days or amputation based on lineage, there was a borderline statistically significant higher mortality among patients with infections caused by Lineage 1 strains compared with Lineage 2 (36% versus 19%, *P* value = 0.05).

**TABLE 2:** Patient characteristics, clinical outcomes, and MARTX toxinotype of Lineage 1 and Lineage 2

Characteristic	Lineage 1 strains (*n *= 61)	Lineage 2 strains (*n *= 48)	*P* value
Patient characteristics			
Age, yrs (avg, range)	61 (12–86)	55 (5–87)	0.49
Male (*n*, %)	52 (85)	44 (92)	0.64
Infection (*n*, %)			
Gastrointestinal	25 (41)	13 (27)	0.12
Wound	29 (48)	28 (58)	0.30
Unspecified	7 (11)	7 (15)	0.37
Severity (*n*, %)			
Hospitalization >14 days	6 (10)	6 (13)	0.65
Amputation	7 (11)	7 (15)	0.37
Death	22 (36)	9 (19)	0.05
Time to death, days (range)	1–59	2–39	
MARTX type (*n*, %)			
C	27 (44)	27 (56)	0.19
M	32 (52)	14 (29)	0.03
E	0	4 (8)	NA^*a*^
D	1 (2)	0	NA
F	0	1 (2)	NA

a NA, not applicable.

### MARTX variants.

**FIG 4:**
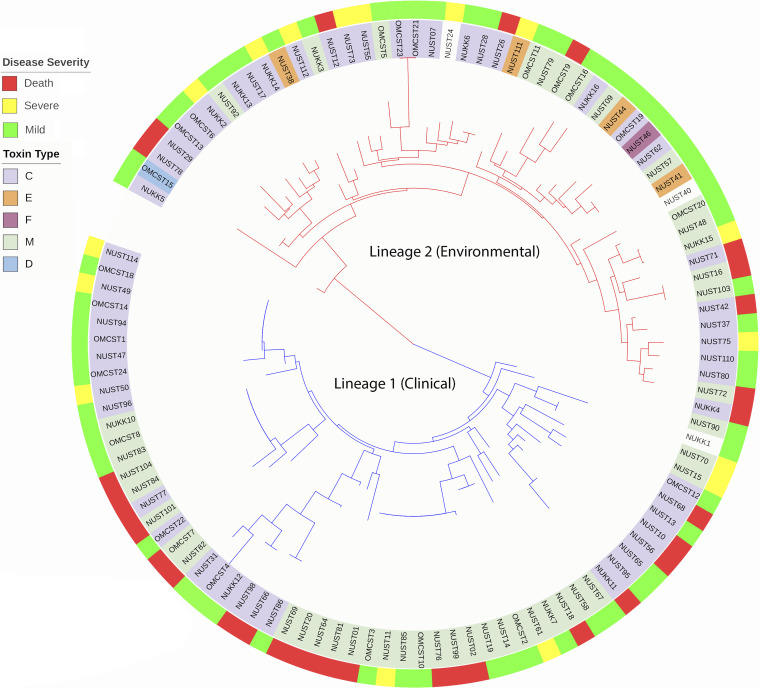
Rooted maximum likelihood circular phylogenetic tree of MLSA sequences Vibrio vulnificus isolate collection. Maximum likelihood phylogenetic analysis based on alignment of concatenated MLSA sequences from 109 isolates sequenced in this study. Tree is midpoint rooted. Isolate name highlight color represents the MARTX toxin type, and the outer colored circle represents the clinical outcome. Interior tree branches represent phylogenetic distance, and color of branches the lineage as indicated.

The multifunctional autoprocessing repeats-in-toxin (MARTX) toxin is the primary virulence factor of V. vulnificus, is highly variable, and can rearrange by homologous recombination, with the M-type frequently arising from a C-type progenitor (24, 25) (Fig. 1). Among the 109 sequenced isolates, MARTX toxinotypes included 54 C-type and 46 M-type. Rare toxinotypes included four E-type, one D-type, and one type found to be shared with previously sequenced isolate FORC-009 and herein referred to as F-type (Table 3). Three of the strains had incomplete toxin genes, and thus the toxinotype could not be assigned. Fig. 4 depicts toxinotype along the phylogenetic tree in the inner ring. Both C- and M-type toxins are found in both Lineages 1 and 2. There was a relative preponderance of type M toxin in Lineage 1 strains, although this was not statistically significant. All four E-type toxins occurred only in Lineage 2 and were from wound infection isolates.

**TABLE 3:** MARTX toxin variant association with severe outcome

Patient Outcomes	C (*n *= 54)	M (*n *= 46)	*P* value (C vs M)	F (FORC) (*n *= 1)	E (*n *= 4)	D (*n *= 1)	Truncated (*n *= 3)
Severe Outcome (*n*, %)	22 (40)	22 (48)	0.35	0	1 (25)	0	1
Hospitalization >14 days	7 (13)	4 (9)	0.41	0	0	0	1 (33)
Amputation	7 (13)	6 (13)	1	0	1 (25)	0	0
Death	15 (28)	16 (35)	0.41	0	0	0	0
Time to death (days)	1–39	1–59					

Of patients with a C- or M-type toxin, 44% had a severe outcome, although there was no statistically significant difference in any adverse outcome based on toxinotype (Table 3). One patient with an E-type toxin had a severe outcome. Neither patient with a strain producing the F- or D-type toxin had a severe outcome.

## DISCUSSION

Vibrio vulnificus is a Gram-negative halophilic bacterium that is part of the natural flora of warm coastal environments but is also an opportunistic human pathogen acquired via ingestion or water exposure leading to a broad range of clinical manifestations, including gastroenteritis, wound infections, and septic shock. Given the overall low prevalence of this infection, studies have been done to identify pathogenic determinants given the high number of humans at risk. In the present study, we assembled the first isolate collection for V. vulnificus with paired clinical data. In total, 109 clinical isolates from across the United States underwent genomic extraction, phylogenetic analysis, and identification of MARTX toxin type. Despite earlier studies suggesting the majority of clinical isolates arise from Lineage 1, we found roughly equal distribution of infections between Lineage 1 and 2. We found a borderline statistically significant higher mortality rate in Lineage 1 isolates. This suggests that the earlier studies may have been biased to predominantly include clinical isolates from patients who had died from their infection, and thus the isolates were more likely to be preserved. However, this is only speculation as all prior studies on phylogeny of V. vulnificus have not included paired analysis of patient outcomes.

Previous studies also have suggested that the strains that secrete the C-type MARTX toxin are more virulent in mice (24, 27). Thus, it was hypothesized that C-type toxin could be associated with more severe patient outcomes. However, we found that C-type and M-type toxins were most frequently present in the clinical isolates but that the MARTX toxin variant type was not predictive of disease severity. Less severe outcomes were associated with E-, F-, and D-type toxins, although these cases are rare in our collection, and thus definitive conclusions regarding disease severity correlation would require a substantially larger collection. If further studies suggest that C-type or M-type toxins are more likely to cause severe disease, public health surveillance programs could potentially use these markers for environmental screening.

Furthermore, a recent study using *V. vulnificus* infection of mice showed no correlation of virulence to genotype or biochemcial assays (9). Our data are also in support of a recent paper that showed no correlation of genotype or standard biochemical assays used to identify bacteria in the clinical setting with virulence potential in mice (15). One recent study has suggested that bacteria might be differentiated by ecotype. This study suggested that strains from the C1 ecotype, which readily degraded a variety of carbohydrates (designated as bloomers), could be more highly virulent (29). A major advantage of our study is that the collection of stains assembled with paired outcome data could be used to further test this possibility as well as other factors that could link growth characteristics with virulence.

Overall, our study of clinical isolates reveals that V. vulnificus from both major phylogenetic lineages are capable of causing severe disease in humans. Of importance for public health surveillance, these data suggest that PCR and sequence-based monitoring of the *vcg* allele to distinguish *vcgC* or *vcgE* or any other method based on distinguishing the two primary lineages in the environment or seafood are not likely to be sufficient to prevent the incidence of severe infections in humans.

## MATERIALS AND METHODS

### Bacterial isolates and identification.

The Centers for Disease Control and Prevention (CDC) and the Food and Drug Administration (FDA) established the Cholera and Other Vibrio Illness Surveillance (COVIS), a national passive surveillance system to monitor the incidence of *Vibrio* infections in the United States (30). All data for this study were de-identified to remove patient identifiers before being released from Ochsner or the CDC. The project was reviewed by the Northwestern University Institution Review Board and declared exempt.

The first pool of bacterial isolates we obtained for this study comprised 73 clinical V. vulnificus isolates with linked clinical information. These isolates were provided to the CDC between January 2007 and December 2013 as part of COVIS. All isolates in this pool were given the designation “NUST#.”

The second pool comprised 40 clinical isolates from the CDC (designated “NUKK#”) or from Ochsner Medical Center (designated “OMC#”), although only 36 had corresponding clinical data sufficient for this analysis. Isolates in the second pool were from an expanded time frame between 2001 and 2019 and were specifically selected to increase representation of isolates from Gulf Coast states. In total, we collected 109 isolates that had corresponding clinical data (Table S1, supplemental data set). All isolates were identified to the species level using two culture methods; V. vulnificus colonies grew as yellow or green on Thiosulfate Citrate Bile Salts Sucrose (TCBS) agar (BD, Franklin Lakes, NJ) and green-blue or turquoise-blue on CHROMagar *Vibrio* (Paris, France). PCR amplification to confirm the *vcg* allele was done for the first pool according to the methods of Rosche et al. (10). The second pool *vcg* alleles were assigned from the *vcg* gene sequence extracted from whole-genome sequencing (10). Final verification of species of the second pool was identified from the whole-genome sequence using ribosomal multilocus sequence typing at https://pubmlst.org/species-id (31).

COVIS data were also obtained from the CDC for a total of 2,431 reported V. vulnificus cases from 2001 to 2019, and data were analyzed alongside the data from isolates with paired clinical data.

### DNA extraction.

Bacterial isolates stored at −80°C were plated on Luria-Bertani (LB) agar and cultured overnight at 37°C. Single colonies were inoculated in LB broth and grown overnight at 37°C with shaking. Genomic DNA was extracted from broth cultures using the Promega Maxwell 16 Cell DNA purification kit (Madison, WI) according to manufacturer's protocol. Genomic DNA extracts were stored at −80°C.

### Whole-genome sequencing and assembly.

For the first pool of isolates, genomic DNA libraries were prepared using the Illumina Nextera XT DNA Library Preparation kit (San Diego, CA). DNA library concentrations were quantified using the Quant-iT dsDNA High-Sensitivity assay kit (Life Technologies, Grand Island, NY). Equal amounts of each library (200 ng) were pooled and sequenced on the Illumina HiSeq 4000 system to generate 150-bp paired-end reads. For the first pool of isolates, sequencing was performed at the University of Maryland School of Medicine Institute for Genome Sciences, Baltimore, MD. Raw sequencing reads were assembled *de novo* using SPAdes v3.10.0 (32) as implemented at the Pathosystems Resource Integration Center (PATRIC) Bioinformatics Resource Center (33) and annotated using the PATRIC RASTtk-enabled Genome Annotation Service (34). For the second pool of isolates, genomic DNA was submitted for 150-Mbp Illumina whole-genome sequencing at the Microbial Genome Sequencing Center (MiGS) in Pittsburgh, Pennsylvania. Species assignment was verified from sequence reads using kraken v1.0 and the minikraken 8GB database (35). Adapter trimming and quality filtering of sequencing reads was performed using Trimmomatic v0.36 (36). Assembly was performed with SPAdes v3.9.1 (32), and assemblies were filtered to remove contigs shorter than 200 bp and with less than 5× average read coverage. We deposited the genomes into GenBank under NCBI BioProject accession no. PRJNA813597. A BioSample was created for each strain with accession numbers SAMN26511979 to SAMN26512087.

### MLSA.

Partial gene sequences for the standardized Vibrio vulnificus 10-locus MLSA method for *glp*, *gyrB*, *mdh*, *metG*, *purM*, *dtdS*, *lysA*, *pntA*, *pyrC*, and *tnaA* were extracted from sequenced genomes. MLSA data for reference genomes were selected from 473 isolates available online at https://pubmlst.org/vvulnificus/ based on representative positioning on the phylogenetic tree publicly available at http://ncbi.nlm.nih.gov/genome/189. MLSA allele sequences from each isolate were concatenated and aligned using mafft v7.310 (37). Maximum likelihood phylogeny was generated from the alignment with iqtree v1.6.1 using the ModelFinder function to estimate the best-fit nucleotide substitution model by means of Bayesian information criterion (BIC) (38, 39). Tree topology was assessed both with the Shimodaira-Hsegawa approximate likelihood ratio test (SH-aLRT) and with the ultrafast bootstrap (UFboot) with 1,000 replicates each (40, 41). Phylogenetic tree was visualized and annotated using the Interactive Tree of Life (iTOL) (42).

### Clinical analysis.

Patient characteristics, clinical presentation, and outcomes were analyzed for all infections reported to COVIS from 2001 through 2019. Severity of infection was categorized as follows: Category 1 was “mild” with recovery; Category 2 was “severe,” defined as requiring hospitalization for >14 days and/or amputation; and Category 3 was death. The unpaired *t* test was used to compare means, and chi-squared analysis was performed to compare proportions using Microsoft Excel.
